# Harnessing Epigenetics: Innovative Approaches in Diagnosing and Combating Viral Acute Respiratory Infections

**DOI:** 10.3390/pathogens14020129

**Published:** 2025-02-01

**Authors:** Ankita Saha, Anirban Ganguly, Anoop Kumar, Nityanand Srivastava, Rajiv Pathak

**Affiliations:** 1 Department of Cell Biology, Albert Einstein College of Medicine, Bronx, New York, NY 10461, USA; ankita.saha@einsteinmed.edu (A.S.); nityanand.srivastava@einsteinmed.edu (N.S.); 2 Department of Biochemistry, All India Institute of Medical Sciences, Deoghar 814152, India; anirban.biochemistry@aiimsdeoghar.edu.in; 3 Molecular Diagnostic Laboratory, National Institute of Biologicals, Noida 201309, India; akmeena87@gmail.com; 4 Department of Genetics, Albert Einstein College of Medicine, Bronx, New York, NY 10461, USA

**Keywords:** virus, epigenetics, acute respiratory infections, viral ARIs, DNA methylation, histone modifications, biomarkers

## Abstract

Acute respiratory infections (ARIs) caused by viruses such as SARS-CoV-2, influenza viruses, and respiratory syncytial virus (RSV), pose significant global health challenges, particularly for the elderly and immunocompromised individuals. Substantial evidence indicates that acute viral infections can manipulate the host’s epigenome through mechanisms like DNA methylation and histone modifications as part of the immune response. These epigenetic alterations can persist beyond the acute phase, influencing long-term immunity and susceptibility to subsequent infections. Post-infection modulation of the host epigenome may help distinguish infected from uninfected individuals and predict disease severity. Understanding these interactions is crucial for developing effective treatments and preventive strategies for viral ARIs. This review highlights the critical role of epigenetic modifications following viral ARIs in regulating the host’s innate immune defense mechanisms. We discuss the implications of these modifications for diagnosing, preventing, and treating viral infections, contributing to the advancement of precision medicine. Recent studies have identified specific epigenetic changes, such as hypermethylation of interferon-stimulated genes in severe COVID-19 cases, which could serve as biomarkers for early detection and disease progression. Additionally, epigenetic therapies, including inhibitors of DNA methyltransferases and histone deacetylases, show promise in modulating the immune response and improving patient outcomes. Overall, this review provides valuable insights into the epigenetic landscape of viral ARIs, extending beyond traditional genetic perspectives. These insights are essential for advancing diagnostic techniques and developing innovative treatments to address the growing threat of emerging viruses causing ARIs globally.

## 1. Introduction

Viral acute respiratory infections (ARIs) remain a significant global health challenge. Major viral pathogens responsible for ARIs include influenza viruses, respiratory syncytial virus (RSV), rhinoviruses, and coronaviruses, including the severe acute respiratory syndrome coronavirus 2 (SARS-CoV-2). ARIs can present with a spectrum of clinical manifestations, ranging from mild respiratory discomfort to severe complications, such as acute respiratory distress syndrome (ARDS), which may result in fatal outcomes. Vulnerable populations, including the elderly, children, and immunocompromised individuals, are particularly susceptible to severe outcomes associated with ARIs. In recent decades, epigenetic studies have provided crucial insights into the molecular mechanisms governing host–pathogen interactions, emerging as a promising field for the development of therapeutic and diagnostic tools against ARIs [1,2].

The term epigenetics refers to heritable changes in gene expression that arise from modifications to chromatin structure without altering the underlying DNA sequence. Epigenetic mechanisms play a crucial role in modulating immune responses to viral infections [3,4]. Among the most studied epigenetic processes are DNA methylation, chromatin remodeling, histone modifications, and non-coding RNA-mediated regulation, all of which are integral components of cellular regulatory networks. These processes are often exploited by pathogens to evade host defenses. For example, hypermethylation of interferon-related genes in host cells has been associated with increased vulnerability to respiratory viruses, such as influenza and SARS-CoV-2. This hypermethylation suppresses the expression of antiviral genes, compromising the host’s immune defenses and facilitating viral persistence [5]. Post-translational modifications of core histones, such as acetylation and methylation, play critical roles in regulating gene expression. Viruses can exploit these modifications to alter host gene expression in ways that enhance viral survival and dampen the host’s antiviral immune response [6,7]. Similarly, epigenetic mechanisms regulated by non-coding RNAs (ncRNAs), including microRNAs (miRNAs), and long non-coding RNAs (lncRNAs), have emerged as crucial post-transcriptional regulators of ARIs [8,9]. Recent studies have demonstrated how viruses such as SARS-CoV-2, influenza, and RSV reprogram the host epigenome to evade immune surveillance and enhance viral replication through these mechanisms [10,11]. Specific alterations in DNA methylation of immunity-related genes in SARS-CoV-2-infected hosts have been reported, potentially suppressing the immune response via epigenetic modifications [12]. Likewise, studies on RSV and influenza reveal that these viruses can induce histone modifications, leading to the downregulation of antiviral genes and facilitating viral persistence and reproduction [13]. In addition to influencing the acute immune responses, viral modulation of the host epigenome can also alter immune memory such that it modulates the host’s susceptibility to reinfection or co-infection with other pathogens. This highlights the profound impact of viral epigenetic reprogramming on both immediate and long-term host immune defenses.

In this review, we comprehensively examine the mechanisms of epigenetic modulation involved in viral ARIs and their impact on the dynamics of the immune response. We explore how viral infections influence the host epigenome, with a particular focus on their roles in disease progression and immune defense. Furthermore, we discuss both current and potential applications of epigenetic insights along with existing limitations and challenges in diagnostics and therapeutics of viral ARIs. Overall, this review provides a thorough exploration of epigenetic modulation in the context of viral ARIs and underscores how recent advances in epigenetics can form the basis for innovative strategies to combat respiratory viral infections, offering novel perspectives on their diagnosis and treatment.

## 2. Mechanisms of Epigenetic Regulation in Viral ARIs

### 2.1. DNA Methylation: Role in Viral Infection and Immune Response

DNA methylation, the covalent addition of methyl groups to cytosine residues within CpG dinucleotides, represents a crucial epigenetic mechanism regulating gene expression without altering the underlying DNA sequence. This modification generally suppresses gene transcription, particularly when located in promoter regions [14]. Respiratory viruses, such as SARS-CoV-2, RSV, and influenza A virus (IAV), have evolved strategies to manipulate host DNA methylation patterns. By modulating these epigenetic landscapes, these viruses can effectively influence host immune responses, promoting viral persistence and evasion of host immune defenses [15,16].

Recent studies reveal that SARS-CoV-2 and RSV infections significantly alter the host’s DNA methylation landscape to evade immune defenses and promote viral persistence. SARS-CoV-2 induces hypermethylation of interferon-stimulated gene (ISG) promoters, suppressing antiviral responses and enhancing viral replication [17,18]. It also hypermethylates genes involved in antigen presentation, such as MHC class I and II, impairing the host’s ability to recognize and eliminate infected cells [19,20]. Similarly, RSV exploits promoter hypermethylation of pro-inflammatory cytokine genes including interleukin-6 (IL-6) and tumor necrosis factor-alpha (TNF-α), reducing their expression and dampening immune responses to support viral replication [21,22]. IAV has also been observed to manipulate host DNA methylation by upregulating DNA methyltransferases (DNMTs), particularly DNMT1, leading to hypermethylation of antiviral genes, including interferon-stimulated genes (ISGs). This suppresses innate immune responses and enhances viral replication [23]. The viral NS1 protein may further recruit DNMTs to the promoters of immune-related genes, creating hypermethylated, transcriptionally repressive regions that enhance viral persistence by reducing interferon signaling [24]. These findings underscore the therapeutic potential of DNMT inhibitors to restore antiviral gene expression and highlight DNA methylation profiling as a biomarker for identifying individuals at higher risk of severe infection outcomes [25,26]. Together with SARS-CoV-2 and RSV, respiratory viruses demonstrate sophisticated strategies to modulate host DNA methylation, shaping immune responses to facilitate viral survival and replication, offering valuable insights into viral pathogenesis and epigenetic-based therapies.

### 2.2. Histone Modifications: Mechanisms and Implications in ARIs

Viruses have evolved sophisticated mechanisms to manipulate host histone modifications, enabling them to evade immune responses, optimize replication conditions, and ensure survival within host cells. Histone acetylation, methylation, and deacetylation are primary targets for viral strategies, with each modification playing a distinct role in immune suppression. By manipulating these epigenetic changes and altering chromatin structure and gene accessibility, viruses reprogram host defenses to support their replication and survival.

Histone acetylation, a key regulator of active gene transcription [27], is frequently targeted by viral proteins. For instance, IAV’s NS1 protein inhibits histone acetyltransferases (HATs), reducing histone H3 and H4 acetylation to suppress antiviral genes [28]. Similarly, RSV decreases histone acetylation by driving histone deacetylase 2 (HDAC2) expression, at interferon-stimulated gene (ISG) promoters, thereby weakening the host’s antiviral response and promoting viral replication [29]. Viruses also exploit histone methylation, a process that can either activate or repress gene expression depending on the specific amino acid residue undergoing methylation [30]. SARS-CoV-2 enhances repressive H3K27me3 methylation at antiviral gene promoters, downregulating their expression to promote viral replication [31]. Histone deacetylases (HDACs) facilitate the removal of acetyl groups from histone proteins, causing chromatin to condense and resulting in the suppression of transcriptional activity. Viruses often exploit HDACs to silence immune genes, thereby ensuring their survival [32,33]. For example, the HIV Tat protein recruits HDAC1 to antiviral gene promoters, thereby repressing immune gene expression and promoting persistent viral infection [34]. Furthermore, HDAC6 has been implicated in regulating the innate immune response during infection, and studies indicate that inhibiting HDAC6 may enhance antiviral immunity [35,36]. These findings highlight the therapeutic potential of targeting HDACs to counter viral immune evasion strategies.

Different viruses exhibit unique strategies in manipulating host histone modifications, which may correlate with their pathogenicity and virulence. For instance, highly pathogenic strains such as H5N1 avian influenza and MERS-CoV enhance repressive H3K27me3 marks while decreasing the activating H3K4me3 marks at ISGs, limiting immune gene expression and promoting immune evasion [37]. In contrast, H1N1 and SARS-CoV increase H3K4me3 and decrease H3K27me3, enhancing ISG expression and facilitating a more balanced interaction with the host’s immune system [31,37]. These patterns of epigenetic modifications reflect the distinct virulence strategies of each virus, highlighting the potential for targeted therapeutic interventions.

### 2.3. Exploitation of Chromatin Remodeling Complexes: Mechanisms and Implications

Chromatin remodeling is a pivotal epigenetic process that modulates gene accessibility by dynamically altering the chromatin structure, thereby regulating transcriptional activity. This highly coordinated reconfiguration of chromatin by large multimeric complexes is indispensable for various cellular processes, including immune responses, particularly during viral infections such as those caused by SARS-CoV-2, influenza, and RSV. Chromatin remodeling complexes, such as SWI/SNF, ISWI (Imitation SWI), chromodomain helicase DNA-binding (CHD), and INO80, utilize the energy derived from ATP hydrolysis to rearrange nucleosomes, allowing controlled DNA access for transcription machinery. This process modulates the accessibility of specific DNA regions to the transcriptional machinery, thereby enabling precise activation or repression of immune-related genes, empowering the host to trigger an antiviral response [38,39,40].

Respiratory viruses manipulate chromatin remodeling to modulate host gene expression, evade immune defenses, and enhance viral replication. SARS-CoV-2, for instance, targets the SWI/SNF complex, a key activator of immune-related genes. By inducing suppressive chromatin states around ISGs, SARS-CoV-2 impairs antiviral signaling pathways, weakening innate immunity and creating a favorable environment for viral replication [41,42]. In SARS-CoV-2 infections, components of the SWI/SNF complex are recruited to the promoters of immune-related genes, where they act as chromatin silencers [43]. By establishing suppressive chromatin configurations around pro-inflammatory cytokines, SARS-CoV-2 dampens antiviral responses, thereby likely facilitating early-stage immune evasion and potentially worsening disease progression [44]. Similarly, RSV also targets SWI/SNF complex to repress interferon-driven genes, redirecting the complex away from antiviral gene loci, thereby improving viral replication efficiency [45].

Beyond its immediate role in modulating immune responses, chromatin remodeling during viral infections can have lasting effects on immune memory [46]. Persistent suppression of immune genes through altered chromatin remodeling can contribute to immune exhaustion, a condition characterized by diminished activity and responsiveness of immune cells during subsequent encounters with pathogens [47,48,49]. In a mouse model of influenza infection, prolonged chromatin-mediated repression of immune gene expression was associated with impaired adaptive immunity upon re-exposure to the virus. This weakened immune response heightened susceptibility to secondary infections, such as bacterial superinfections, which are common complications of viral ARIs [50,51]. These findings underscore the dual impact of chromatin remodeling in shaping both the immediate and long-term dynamics of the host immune defense.

### 2.4. Non-Coding RNAs: Roles of MicroRNAs and Long Non-Coding RNAs in ARIs

Non-coding RNAs (ncRNAs), including miRNAs, and lncRNAs, have emerged as crucial regulators of innate immunity, exerting significant effects on host responses to viral infections at the epigenetic level. These ncRNAs modulate immune responses by either promoting or suppressing viral replication, depending on their interactions with specific cellular pathways. They achieve this by recruiting histone-modifying proteins, blocking the binding of transcription factors, or directly targeting viral genes. Through these mechanisms, ncRNAs play a crucial role in maintaining the intricate balance between viral evasion strategies and host immune defenses [52,53]. MicroRNAs are short, non-coding RNA molecules, usually 21–23 nucleotides long, that regulate gene expression by binding to complementary sequences on target mRNAs, leading to their degradation or translational suppression [54]. In ARIs, miRNAs play a pivotal role in immune regulation by modulating pathways involved in interferon signaling, cytokine production, and antiviral gene expression. For example, miR-155 is often upregulated during viral infections, where it plays a critical role in regulating immune genes and thereby enhancing the host’s antiviral response [55]. Respiratory viruses, including influenza and SARS-CoV-2, actively exploit host miRNAs to facilitate their survival and replication. SARS-CoV-2 infection, for instance, alters the expression of specific miRNAs to weaken immune responses. It has been observed that SARS-CoV-2 upregulates miR-146a, a miRNA that represses pro-inflammatory signaling pathways, effectively dampening immune activation and allowing the virus to replicate with reduced resistance. Similarly, SARS-CoV-2 upregulates miR-21, which inhibits the activity of ISGs that are critical for antiviral defense mechanisms [56,57]. Other respiratory viruses, such as the influenza virus, also employ miRNAs to suppress ISG activity, thereby facilitating viral replication and delaying immune recognition [58,59]. These viral strategies, involving miRNA modulation, exemplify a common mechanism by which respiratory viruses subvert host immunity to establish infection.

Long non-coding RNAs (lncRNAs), typically exceeding 200 nucleotides in length, play critical roles in chromatin remodeling, gene transcription, and immune signaling. They act as molecular scaffolds, decoys, and guides for various molecules, influencing a wide range of cellular processes [60]. In recent years, lncRNAs have gained increasing recognition for their involvement in antiviral immune responses, particularly in the modulation of immune signaling pathways during viral infections [61]. In the context of SARS-CoV-2 infection, several specific lncRNAs are upregulated to modulate immune functions. NEAT1, a well-studied lncRNA, is highly expressed in SARS-CoV-2-infected cells and promotes the formation of paraspeckles, subnuclear structures that may be involved in cytokine regulation. NEAT1’s activity in these cells may help control immune signaling by sequestering immune-related molecules, thereby indirectly influencing cytokine production during infection [55,62,63].

## 3. Epigenetic Regulation of Innate Immune Responses and Viral Immune Evasion

**Figure 1:**
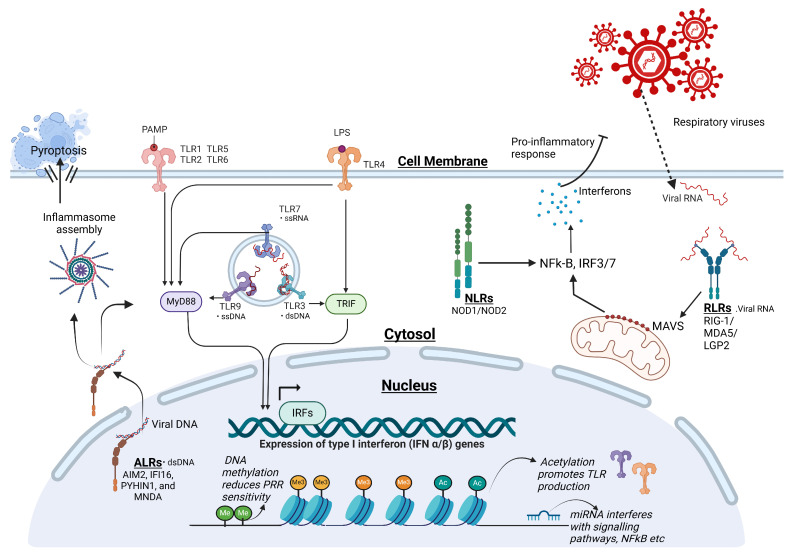
**Epigenetic regulation of innate immune defense in response to respiratory viral infections**: This schematic illustrates the mechanisms of innate immune defense pathways and their regulation by epigenetic modifications during acute respiratory viral infections. Toll-like receptors (TLRs) at the cell membrane and within endosomes detect pathogen-associated molecular patterns (PAMPs), with TLR2/4 responding to viral proteins and endosomal TLR3, TLR7/8, and TLR9 recognizing viral dsRNA, ssRNA, and CpG DNA, respectively. These receptors activate adaptor proteins such as MyD88 and TRIF, triggering NF-κB and IRF3/7 signaling pathways that drive the production of pro-inflammatory cytokines and type I interferons (IFN-α/β). In the cytosol, RIG-I-like receptors (RLRs), including RIG-I and MDA5, detect viral RNA and signal through mitochondrial antiviral signaling protein (MAVS) to activate NF-κB and IRFs. NOD-like receptors (NLRs) such as NOD1 and NOD2 sense bacterial and viral components, promoting NF-κB activation and RNA degradation via OAS2 and RNase L, while absent in melanoma 2-like receptors (ALRs) like AIM2 detect viral dsDNA, forming inflammasomes that activate IL-1β and IL-18. Epigenetic regulation in the nucleus modulates these immune responses, with DNA methylation suppressing PRR gene expression, histone acetylation (e.g., H3K27ac) enhancing gene expression, and miRNAs interfering with pathways like NF-κB (Created with BioRender.com).

Innate immune cells possess sophisticated mechanisms to detect harmful pathogens, including viruses, and play a crucial role in initiating protective immunity as the body’s first line of defense. These mechanisms, regulated at various levels through epigenetic controls, quickly trigger immune responses during viral ARIs. Pattern recognition receptors (PRRs) in various immune and non-immune cells, such as monocytes, dendritic cells, NK cells, and epithelial cells, identify conserved microbial patterns to detect viral infections [64]. Unlike the antigen-specific receptors on T and B cells, PRRs—such as Toll-like, RIG-I-like, NOD-like, AIM2-like, and C-type lectin receptors—function at cell surfaces or within cells to sense pathogens [65,66,67,68] (Figure 1).

### 3.1. Toll-like Receptors (TLRs)

TLRs are key PRRs involved in detecting pathogen-associated molecular patterns (PAMPs) from viruses, bacteria, and fungi. TLRs initiate antiviral immune responses by producing cytokines and chemokines. Various TLRs (e.g., TLR2/1, TLR2/6, TLR3, TLR4, TLR5, TLR7, TLR8, TLR9, and TLR11) are localized to the cell membrane or endosomes to recognize bacterial and viral molecules like lipopeptides, lipopolysaccharides, flagellin, DNA, and RNA via a conserved leucine-rich repeat (LRR) structure linked to an intracellular Toll/IL-1 receptor (TIR) domain [69,70]. Human TLRs sensing viral nucleic acids, such as TLR3, TLR7, TLR8, and TLR9, are primarily located in endosomes, while those targeting bacterial components reside on the cell membrane. TLR3 recognizes viral dsRNA through the TRIF adaptor, TLR7 and TLR8 detect ssRNA via MyD88, and TLR9 identifies unmethylated CpG DNA [71]. Additionally, viral proteins activate inflammatory responses through TLR2 and TLR4. Emerging RNA viruses, including SARS-CoV, MERS-CoV, and SARS-CoV-2, are recognized by TLR2, TLR3, TLR4, TLR7, TLR8, and other PRRs, leading to immune activation [72]. TLR activation depends on adaptor proteins like MyD88 (used by all TLRs except TLR3) and TRIF (specific to TLR3 and TLR4). Additional adaptors, such as TIRAP, TRAM, and SARM, also contribute to TLR signaling. This activation triggers intracellular pathways, including NF-κB, MAPK, and interferon regulatory factor 3 (IRF3), culminating in the production of proinflammatory cytokines and type I interferons essential for antiviral immunity [68,73,74].

### 3.2. RIG-I-like Receptors (RLRs)

Cells utilize not only TLRs but also RLRs to detect viral RNA and initiate antiviral responses, which are crucial for interferon (IFN) production during RNA virus infections. The RLR family includes RIG-I, MDA5, and LGP2, all featuring a helicase domain and a C-terminal domain (CTD) for viral RNA binding. RIG-I and MDA5, unlike LGP2, also possess caspase activation and recruitment domains (CARDs) that interact with the adaptor protein MAVS. This interaction with MAVS activates downstream signaling proteins, including TRAF3/6 and IKK family members (IKKε, IKKα/β, and TBK1), culminating in IRF-3/7 and NF-κB activation, which drive IFN production and pro-inflammatory responses [75,76]. RIG-I recognizes viral RNA with a 5′-triphosphate or 5′-diphosphate group and a panhandle double-stranded structure. Upon RNA binding, RIG-I utilizes ATP to release its CARDs, which subsequently interact with MAVS on mitochondria. This interaction triggers the formation of filamentous RIG-I structures along the RNA, allowing the CARD domains to adopt a helical configuration that facilitates MAVS recruitment of additional signaling proteins to initiate the antiviral response [76,77]. Knockout (KO) studies have revealed RIG-I’s critical role in viral detection across diverse cell types [78]. However, MDA5 specializes in recognizing longer dsRNAs and plays a vital role in detecting viruses from the *Picornaviridae* family, as evidenced by MDA5-KO mouse studies, though it can recognize other viral species as well [79].

LGP2, a member of the RLR family, shares structural similarity with RIG-I and MDA5 but lacks CARDs, preventing it from directly initiating signaling through MAVS. This distinction sets LGP2 apart functionally, as it primarily acts as a modulator of the RLR pathway, influencing IFN production and other immune responses by either enhancing or suppressing antiviral signaling. LGP2 plays a crucial role in MDA5-dependent recognition of picornaviruses, such as encephalomyocarditis virus (EMCV) [76,80]. Additionally, LGP2 contributes to regulating RNA interference by interacting with Dicer, an essential enzyme for miRNA processing, suggesting a potential role in indirectly modulating antiviral gene expression. LGP2 can also bind to the transactivation response RNA-binding protein (TRBP), which is a positive regulator of Dicer activity, thereby affecting miRNA maturation. Through these interactions, LGP2 influences gene expression and promotes caspase-dependent apoptosis under certain conditions [81,82]. In the context of West Nile virus (WNV) infection, LGP2 plays a vital role in CD8^+^ T-cell survival and functionality, though it does not directly participate in MAVS-mediated IFN production [83]. These results emphasize the multifaceted roles of LGP2 in antiviral immunity and underscore the importance of further investigation to clarify its broader roles.

### 3.3. NOD-like Receptors (NLRs)

NOD-like receptors (NLRs), part of the nucleotide-binding domain and leucine-rich repeat (NBD-LRR) family, serve as essential cytosolic pattern recognition receptors in the host’s antiviral immune response. Among the regulatory NLRs, NOD1 and NOD2 enhance pro-inflammatory pathways by forming a multiprotein complex called the NODosome, which significantly influences interferon (IFN) production and NF-κB signaling pathways in response to viral infections [84,85].

NOD1 regulates antiviral responses against specific single-stranded RNA viruses, such as hepatitis C virus, and double-stranded DNA viruses, such as human cytomegalovirus (CMV). However, NOD1 does not universally detect all viruses, as it fails to sense single-stranded RNA viruses like vesicular stomatitis virus (VSV) and RSV. NOD1 enhances MDA5/MAVS-mediated immune signaling pathways, and this interaction is crucial for robust innate antiviral responses. Notably, MDA5’s ability to bind viral RNA in collaboration with NOD1 is conserved across species. Furthermore, the activation of NOD1 in lung epithelial cells by agonists such as TriDAP induces an antiviral state, effectively inhibiting SARS-CoV-2 replication [86].

NOD2, a cytosolic pattern recognition receptor, is significantly upregulated in the host’s transcriptional response to various pathogens. It is predominantly expressed in macrophages, neutrophils, dendritic cells, and bronchial epithelial cells, where it recognizes components from both bacterial and viral pathogens. Upon recognition of these microbial components, NOD2 activates NF-κB signaling, leading to the transcription of numerous innate immune response genes involved in pathogen defense. Studies have demonstrated that NOD2 detects several respiratory bacteria and recognizes RSV’s single-stranded RNA. This recognition activates the interferon regulatory factor 3 (IRF3) signaling pathway, which is crucial for antiviral immunity. Furthermore, NOD2 interacts with 2′,5′-oligoadenylate synthetase type 2 (OAS2), facilitating the activation of RNase L, which degrades viral RNA, thereby limiting viral replication. In addition to its role in pathogen recognition, NOD2 signaling relies on receptor-interacting serine-threonine kinase 2 (RIPK2), a critical adaptor protein in the NLR signaling pathway. RIPK2 is upregulated during infections and plays an essential role in activating both the NF-κB and MAPK signaling pathways, which drive pro-inflammatory gene expression. Additionally, RIPK2 contributes to apoptosis, an essential process in the elimination of infected cells, further highlighting its importance in innate immune responses [87,88].

### 3.4. Absent in Melanoma 2 (AIM2)-like Receptors (ALRs)

Absent in melanoma 2 (AIM2)-like receptors (ALRs) are a family of PRRs that detect intracellular dsDNA via their HIN-200 domain, which directly binds to DNA. Their pyrin domain (PYD) interacts with the adaptor protein ASC, facilitating inflammasome assembly, which triggers the proteolytic activation of pro-inflammatory cytokines such as IL-1β and IL-18 [89]. Among ALRs, IFI16 is predominantly expressed in vascular endothelial cells, keratinocytes, and hematopoietic cells. Upon sensing foreign DNA, IFI16 translocates from the nucleus to the cytoplasm. There, it promotes type I interferon (IFN) production, the release of inflammatory cytokines, and programmed cell death. Notably, IFI16 stabilizes cyclic GMP-AMP synthase (cGAS) activity, enhancing its interaction with viral DNA. This leads to the activation of the STING pathway, which in turn triggers IRF3-mediated production of IFN-β during viral infections [90,91]. Interestingly, IFI16 has been detected within HCMV virions during their egress and in the cytoplasm of Kaposi’s sarcoma-associated herpesvirus (KSHV)-infected cells, where it is packaged into exosomes and subsequently released extracellularly. Emerging evidence suggests that extracellular IFI16 has significant pro-inflammatory activity. Specifically, it induces cytokine secretion in endothelial cells through a MyD88-dependent Toll-like receptor (TLR) pathway. This results in the release of pro-inflammatory mediators, including IL-6, IL-8, CCL2, CCL5, and CCL20 [92,93,94,95].

### 3.5. C-Type Lectin Receptors (CLRs)

Innate immune cells, such as monocytes, macrophages, dendritic cells (DCs), and Langerhans cells (LCs), express C-type lectin receptors (CLRs), a class of PRRs. CLRs are typically classified into two major groups, namely Group I, which includes the mannose receptor family, and Group II, which comprises receptors such as Dectin-1 (CLEC7A) and DCIR (CLEC4A). These receptors often contain specific signaling motifs like immunoreceptor tyrosine-based activation motifs (ITAMs) or inhibitory motifs (ITIMs). However, some CLRs, including DC-SIGN (CD209) and DEC-205, lack these signaling motifs [96,97,98]. CLRs recognize carbohydrate structures via conserved carbohydrate recognition domains (CRDs) in a calcium-dependent manner, which is crucial for pathogen detection and binding. This recognition facilitates high-affinity ligand interaction, leading to internalization and subsequent pathogen degradation. Degradation frequently occurs through lysosomal pathways, as exemplified by DC-SIGN and DEC-205, or through autophagy, as seen with langerin [96,98]. The functional outcomes of CLR-mediated pathogen recognition are influenced by the specific CLR and the type of innate immune cell involved.

In viral infections, CLRs exhibit diverse roles, with some receptors promoting antiviral defenses through signaling and pathogen degradation, while others are hijacked by viruses to facilitate replication or evade immune detection. For instance, DC-SIGN mediates endocytosis of HIV-1, facilitating viral dissemination, while MDL-1 (CLEC5A) induces pro-inflammatory responses during Dengue virus infection. Certain CLRs, such as BDCA-2 and DCIR, suppress type I IFN responses, critical for robust antiviral immunity. DC-SIGN and its homolog L-SIGN enhance infection by viruses like SARS-CoV-2 and HIV-1. Upon HIV-1 binding to DC-SIGN, signaling pathways modulate TLR-mediated responses. The degradation of HIV-1 in endosomes triggers TLR8-dependent NF-κB activation, driving transcription of integrated viral DNA. Moreover, DC-SIGN mediates NF-κB phosphorylation, promoting HIV-1 protein production and replication, while simultaneously inhibiting type I IFN responses. This immune evasion strategy is shared by viruses such as measles virus (MV), which disrupts RIG-I and MDA5 activation through phosphatase inhibition. Additionally, CLRs such as the mannose receptor (MR) and langerin engage with shared signaling pathways, including lymphocyte-specific protein 1 (LSP1). The MR inhibits TLR4-induced IL-12 production, while ITIM-bearing receptors like DCIR and MICL suppress IL-12 secretion [97,99].

DCIR, found on macrophages and dendritic cells, exerts an inhibitory effect on the immune response by reducing the production of pro-inflammatory cytokines, including IL-1β and IL-6, when triggered by CpG-ODN stimulation. Although DCIR reduces IFNα production, it sustains type I IFN signaling, as evidenced by diminished STAT1 phosphorylation in DCIR-deficient dendritic cells during *Mycobacterium tuberculosis* infection. This suggests DCIR is involved in maintaining STAT1 activity and type I IFN signaling. Additionally, DCIR suppresses IL12p70 production and impairs TH1 differentiation, although these effects vary depending on the dendritic cell subset and the pathogen. The role of DCIR in modulating type I IFN responses during viral infections in humans remains unclear. In humans, DCIR facilitates HIV-1 capture and dissemination, promoting viral replication and entry, particularly by upregulating its expression on T cells. In mice, DCIR enables the internalization of Chikungunya virus and appears to play a protective role during infection, illustrating its context-dependent functionality. However, whether DCIR acts as a pattern recognition receptor (PRR) to enhance type I IFN signaling in these contexts remains to be conclusively demonstrated [96,98].

BDCA-2 (CLEC4C or CD303), a C-type lectin receptor primarily expressed on plasmacytoid dendritic cells (pDCs), serves as a key marker for these cells [100]. Activation of BDCA-2 by stimuli such as CpG oligonucleotides, Influenza virus, or DNA-autoantibody complexes suppresses type I IFN responses in pDCs. Crosslinking BDCA-2 with specific antibodies inhibits type I IFN and pro-inflammatory cytokine production downstream of TLR7 and TLR9 activation. Additionally, BDCA-2 interacts with the Hepatitis C Virus (HCV) glycoprotein E2, further suppressing type I IFN responses. This inhibition occurs through interference with TLR signaling pathways, including the prevention of MyD88 recruitment, which blocks TLR-mediated IL-6 production [101,102].

Myeloid-expressed CLRs detect viral glycoproteins, initiating protective immune responses such as viral uptake, degradation, and antigen presentation. For example, CD207 (langerin) on Langerhans cells binds HIV glycoproteins, facilitating their degradation via a TRIM5α-dependent autophagy pathway. This process transfers antigens to dendritic cells, enabling cross-presentation and subsequent activation of protective T cell responses [103]. Similarly, CLEC9A promotes cross-presentation through phagosomal rupture, which is crucial for controlling infections caused by herpes simplex virus and vaccinia virus [104,105]. In addition to their role in cellular immunity, CLRs contribute to complement-mediated pathogen clearance. The complement system, activated through classical, lectin, or alternative pathways, leads to the deposition of C3b on pathogens, enhancing opsonization and phagocytosis. The soluble CLR mannose-binding lectin (MBL) binds carbohydrate structures like mannose and fucose on pathogens, including HIV, dengue virus (DENV), and SARS-CoV. Acting as an opsonin, MBL triggers complement activation to promote pathogen clearance [96,106]. However, while MBL binds HIV-1 virions and enhances viral clearance, its role in HIV pathogenesis remains uncertain. Complement-mediated opsonization of HIV-1 can enhance viral uptake by dendritic cells and macrophages, potentially facilitating viral spread [107].

## 4. Impact of Viruses Associated with Viral ARIs on Host Epigenome

Epigenetic modifications, critical for maintaining genome stability and cellular function, are exploited by viruses to regulate key aspects of their life cycle, including replication, persistence, and transmission [108]. These modifications create a coordinated interaction between viral and cellular factors, favoring viral gene expression while suppressing host cellular genes, thereby influencing immune responses and enhancing host cell susceptibility [109]. Here, we explored viruses associated with viral ARIs that induce epigenetic changes in host cells and their implications in disease progression (Table 1).

### 4.1. Influenza

Influenza is a persistent respiratory infectious disease in humans, causing 300,000–500,000 fatalities annually worldwide. Following the initial pandemic waves, influenza viruses transitioned into seasonal infections, driven by antigenic drift resulting from the error-prone viral polymerase. This process generates mutations that enable the virus to evade immune responses in exposed populations [110]. Reassortment events between derivatives of the 1918 virus and avian influenza viruses led to the emergence of the H2N2 virus during the 1957 Asian influenza pandemic and the H3N2 virus during the 1968 Hong Kong pandemic. These viruses, characterized by their eight gene segments—PB1, PB2, PA, HA, NP, NA, M, and NS—originated from distinct sources. In 1977, the H1N1 “Russian influenza” virus emerged, showing substantial genetic similarity to H1N1 strains from the early 1950s. Unlike its predecessors, this virus did not replace the circulating H3N2 virus. However, in 2009, a novel H1N1 virus, originating from swine and involving reassortment of gene segments from multiple sources, replaced the seasonal H1N1 virus while H3N2 continued to circulate [111,112]. The replication and evolution of influenza viruses relies heavily on host factors. While certain host components are essential for viral replication, restrictive host factors can limit infection. Variability in these host factors across species drives host-adaptive evolution, shaping the virus’s ability to infect and persist in new hosts [112].

The IAV’s nucleoprotein (NP) binds to the viral RNA genome, serving a role analogous to eukaryotic histones binding to DNA. NP undergoes various post-translational modifications essential for its function. Phosphorylation of NP prevents oligomerization, thereby modulating ribonucleoprotein (RNP) activity and viral growth [113,114]. NP also regulates the intracellular localization of RNP and itself through interactions with importin-α [115,116], with SUMOylation and phosphorylation further controlling its nuclear-cytoplasmic transport [117,118]. Ubiquitination and deubiquitination of NP also influence viral genome replication [119,120]. Studies identified acetylation of eight lysine residues on NP, with three specific sites shown to impact viral replication in HEK293T cells expressing the cAMP-response element binding protein (CREB) [121]. Hatakeyama et al. demonstrated that influenza virus nucleoprotein (NP) is acetylated by host acetyltransferases GCN5 and PCAF, influencing viral polymerase activity. Mass spectrometry identified Lys-31 and Lys-90 as acetylation sites, with PCAF and GCN5 exhibiting opposing effects on polymerase activity. These findings highlight the regulatory role of NP acetylation in IAV’s replication [122]. Previous studies show that IAV’s NP interacts with HDAC1, reducing acetylation at lysine 103, a site critical for viral replication. Mutations at this site (K103A and K103R) enhanced viral replication, while HDAC1 facilitated NP nuclear retention and suppressed the TBK1-IRF3 pathway, identifying HDAC1 as a potential antiviral target [123]. The nonstructural protein 1 (NS1) of IAV binds to DNMT3B, mislocalizing it to the cytosol and preventing the methylation of promoters for JAK-STAT signaling suppressor genes. This reveals NS1 as a key driver of epigenetic dysregulation, uncovering a novel mechanism of immune evasion during IAV infection [24].

N (6)-methyladenosine (m(6)A) modification also plays a dual role in regulating host responses during IAV infection [124,125]. TBK1 enhances METTL3 activity, boosting IRF3 production and antiviral immunity, while METTL3-mediated m6A methylation of IFN-β mRNA reduces its stability, suppressing type I interferon responses and promoting viral replication. Depleting METTL3 or YTHDF2 restores IFN-β levels, inhibiting viral gene expression [126,127]. Additionally, some studies suggest the role of miRNAs in IAV infection. miRNA profiling in A549 cells infected with H5N1 and H1N1 strains revealed differential expression, with miR-21-3p notably downregulated. miR-21-3p targets and represses HDAC8, promoting viral replication and mimicking the effects of HDAC8 knockdown. This suggests that miR-21-3p downregulation is part of the host defense response to IAV [128].

### 4.2. Respiratory Syncytial Virus (RSV)

Respiratory syncytial virus (RSV) is one of the major respiratory pathogens and a primary cause of hospitalizations among infants worldwide. It significantly contributes to the global burden of acute lower respiratory infections, including bronchiolitis and pneumonia, resulting in substantial morbidity and mortality among young children [129]. RSV induces epigenetic modifications to evade immune responses, affecting critical signaling pathways in respiratory epithelial cells, such as tyrosine kinase growth factor signaling, the hexosamine biosynthetic pathway (HBP), and extracellular matrix (ECM) secretion. These modifications, including chromatin remodeling and increased gene accessibility, contribute to airway remodeling via TGF-β, ECM, and HBP pathways, potentially leading to chronic respiratory conditions [130,131]. A study on children under 2 years with RSV infection revealed that those developing respiratory sequelae had higher proportions of NK and CD8^+^ T cells than those who fully recovered. Over 5000 differentially methylated positions (DMPs) were identified in these patients, with significant hypomethylation linked to overexpression of genes driving airway inflammation, highlighting epigenetic contributions to recurrent wheezing and asthma [132]. In another study, Elgizouli et al. reported methylation changes in the perforin-1 (PRF1) enhancer, a gene critical for antiviral immunity, following severe RSV infection [133]. Additionally, Wang et al. demonstrated RSV-induced upregulation of the NODAL gene in bronchial epithelial cells (BECs), which is typically hypermethylated in healthy BECs [134]. RSV-induced epigenetic modifications skew T cells toward Th2 and Th17 phenotypes. In murine models, RSV infection upregulated demethylase genes Kdm5b and H3K4 in dendritic cells, suppressing type I IFN and cytokine transcription, leading to reduced pro-inflammatory responses and a Th2-skewed phenotype. KDM5B, a key epigenetic regulator, inhibited innate cytokine production, including IFN-β, in RSV-infected dendritic cells. The inhibition of Kdm5b increased levels of IFN-β, IL-6, and TNF-α, while Kdm5b-deficient mice showed increased IFN-γ, reduced Th2 cytokines, and decreased lung inflammation. Similarly, KDM5B inhibition in human dendritic cells enhanced innate cytokine production and reduced Th2 responses, identifying KDM5B as a potential therapeutic target to enhance antiviral immunity and mitigate RSV pathology [135]. Additionally, methylation of H3K4 by SMYD3 in regulatory T cells plays a critical role in controlling lung inflammation post-RSV infection [136]. These findings suggest RSV induces long-lasting epigenetic modifications that impair immune responses and may contribute to chronic respiratory conditions.

### 4.3. Human Rhinovirus (HRV)

Human rhinovirus (HRV) infection plays a significant role in exacerbating asthma symptoms and is believed to contribute to the development of asthma during early childhood. HRV, a single-stranded RNA virus within the *Picornaviridae* family, encompasses over 160 recognized variants, classified into three species, namely HRV-A, HRV-B, and HRV-C [137]. Among these, HRV-A and HRV-C are strongly associated with lower respiratory tract infections (LRTIs) in children, leading to increased morbidity. Individuals with atopic asthma exhibit heightened susceptibility to HRV infection, experiencing not only a greater frequency of symptomatic respiratory tract infections but also prolonged and more severe respiratory symptoms following infection [138]. Martin Pech and colleagues conducted a study to examine the effects of HRV infection on DNA methylation and mRNA expression in epithelial cells derived from asthmatic and non-asthmatic children. Using primary nasal epithelial cells infected with Rhinovirus-16 (RV-16), they performed genome-wide analyses of DNA methylation and mRNA expression. The study identified 471 CpG sites associated with 268 genes that displayed asthma-specific alterations in response to HRV infection. Among these, 16 CpG sites, including those linked to HLA-B-associated transcript 3 (BAT3) and Neuraminidase 1 (NEU1), were implicated in modulating the host immune response to HRV infection. The findings revealed that HRV infection induces distinct DNA methylation changes that influence mRNA expression in asthmatic children, particularly in genes involved in immune regulation and asthma pathogenesis. These results highlight HRV’s potential contribution to asthma persistence and progression, offering insights into possible therapeutic targets for future interventions [139].

### 4.4. Human Metapneumovirus (HMPV)

Human metapneumovirus (HMPV) is a negative-sense RNA virus belonging to the family *Paramyxoviridae*, subfamily *Pneumovirinae*, and genus *Metapneumovirus*. It is classified into two primary lineages, A and B, which are further subdivided into sublineages A1, A2, B1, and B2 based on variations in surface glycoproteins and antigenic properties [140,141]. Recent studies have highlighted the significance of N6-methyladenosine (m6A) modifications in the HMPV genome. Lu et al. demonstrated that m6A modifications are present in the HMPV genome, antigenome, and mRNA, with the G gene exhibiting the highest m6A peak. The study revealed that m6A modifications enhances viral replication and gene expression, while its depletion reduces the severity of HMPV infection in cell cultures. Additionally, overexpression of m6A-binding proteins significantly increased viral replication, protein synthesis, and RNA levels, underscoring the pro-viral role of m6A in HMPV pathogenesis [142]. These findings establish m6A as a critical factor in HMPV pathogenesis, offering potential avenues for therapeutic intervention.

### 4.5. Adenovirus

Human adenoviruses (HAdVs) are small, non-enveloped DNA viruses that are highly prevalent in the human population. These viruses encode various proteins that mimic functions found in other viruses. One such protein, protein VII, is a small, positively charged molecule that binds to viral DNA in a non-specific manner [143]. Similarly to histones, protein VII plays a crucial role in condensing the viral genome for packaging. It is essential for protecting the viral DNA from damage and regulating the expression of viral genes. Notably, protein VII interacts with cellular chromatin, despite sharing limited sequence similarity with the cellular histone H3 [144,145].

Avgousti et al. demonstrated that protein VII forms complexes with nucleosomes, thereby restricting DNA accessibility and altering the protein composition of host chromatin. Posttranslational modifications of protein VII facilitate its chromatin localization, enabling it to sequester high-mobility group proteins (HMGB1, HMGB2, and HMGB3). Notably, protein VII binds directly to HMGB1, a protein released during inflammatory responses to activate immune signaling, thereby preventing its release. In a mouse model, expression of protein VII in the lungs reduced inflammation-induced HMGB1 levels and inhibited neutrophil recruitment in bronchoalveolar lavage fluid. These findings highlight a viral strategy by which adenoviruses bind nucleosomes and modulate immune signaling, effectively suppressing host inflammatory responses [146]. In another study, Ferrari et al. investigated the role of the adenovirus small E1A protein in promoting oncogenic transformation through its interaction with host lysine acetylases, p300/CBP, and the tumor suppressor RB. Their study found that E1A displaces RB from the E2F transcription factors, enabling p300 to acetylate RB1 at specific lysine residues (K873/K874). This acetylation stabilizes RB1 in a repressive conformation, enabling it to recruit chromatin-modifying enzymes and form p300-E1A-RB1 complexes. These complexes preferentially target and condense chromatin at host genes with high p300 activity within the gene body, including genes involved in TGF-β, TNF, and interleukin signaling pathways. These complexes condense chromatin through mechanisms dependent on histone deacetylase (HDAC) activity, p300 acetylase, and specific acetylation of RB and E1A, thereby repressing host genes that could inhibit viral replication [147]. We have summarized the individual viral proteins from each of the mentioned viruses and their interactions with the host epigenetic machinery, as shown in Table 2.

### 4.6. Severe Acute Respiratory Syndrome Coronavirus 2 (SARS-CoV-2)

Coronaviruses are a family of single-stranded RNA viruses primarily associated with respiratory infections in humans and animals. Seven species of coronaviruses are known to infect humans, with SARS-CoV-2 being the most recent, first identified in Wuhan in December 2019, and the virus responsible for causing COVID-19. Out of these seven species, HCoV-229E, HCoV-NL63, HCoV-OC43, and HCoV-HKU1 typically cause mild respiratory illnesses, whereas MERS-CoV, SARS-CoV, and SARS-CoV-2 are associated with a spectrum of clinical outcomes. These range from mild upper respiratory symptoms to severe, life-threatening conditions such as acute lung injury (ALI) and ARDS [148,149].

SARS-CoV-2 is an enveloped virus with a single-stranded positive-sense RNA genome ranging from 29.7 kilobases (kb) to 29.9 kb [150]. The virus utilizes the angiotensin-converting enzyme 2 (ACE2) receptor, encoded by the ACE2 gene, for cellular entry. This receptor is expressed in various cell types, including lung epithelial cells, and gastrointestinal cells [151,152]. Epigenetic mechanisms significantly influence the regulation and expression of ACE2. Overexpression of ACE2 has been linked to severe COVID-19 outcomes, with HDAC enzymes modulating epigenetic responses that contribute to pro-inflammatory cytokine storms during infection [153]. Conversely, histone deacetylase inhibitors (HDACi) have been shown to reduce ACE2 expression, potentially mitigating disease progression [154]. DNA methylation also significantly impacts ACE2 regulation, with lung epithelial cells exhibiting the lowest levels of DNA methylation across tissues, correlating with high ACE2 expression in these cells [155]. In children, differential methylation of the ACE2 gene at 15 CpG sites has been associated with age, sex, and race, potentially explaining disparities in COVID-19 susceptibility and severity. For instance, females and Black males exhibit lower DNA methylation levels at these sites. Additionally, longer DNA methylation telomere lengths correlate with higher ACE2 methylation in both sexes [156]. These findings highlight the significance of epigenetic variations, particularly DNA methylation signatures in ACE2, in influencing individual susceptibility and the clinical outcomes of SARS-CoV-2 infection.

A study by Mao et al. investigated blood epigenetic alterations, particularly DNA methylation, in 133 young adults with mild/asymptomatic SARS-CoV-2 infection. While most gene expression changes normalized after viral clearance, some methylation sites remained altered for months, resembling patterns seen in autoimmune or inflammatory diseases. Methylation-based machine learning models are being developed to distinguish pre-, during-, and post-infection states, enabling accurate predictions of infection timelines. Early or pre-infection methylation profiles effectively predicted clinical outcomes and mirrored post-infection epigenetic changes. Notably, the post-acute SARS-CoV-2 epigenetic landscape was antiprotective, potentially increasing susceptibility to further infections rather than providing immunity [157]. Kee et al. identified a novel mechanism by which SARS-CoV-2 disrupts host cell epigenetic regulation through histone mimicry. The viral ORF8 protein, containing an ARKS motif critical for histone post-translational modifications (PTMs), interacts with histones, and the nuclear lamina. ORF8 undergoes acetylation, mimicking histone modifications, which disrupts key histone PTMs and induces chromatin compaction. These effects are dependent on the presence of the histone mimic motif, as its absence abolishes chromatin disruption. Importantly, deletion of ORF8 or its mimic motif reduces viral replication and modulates the host transcriptional response, highlighting ORF8’s critical role in viral replication and pathogenesis [158]. Furthermore, a SARS-CoV-2 variant isolated in Singapore with a deletion in the ORF8 gene was associated with milder infections and an enhanced interferon response in patients [159].

RNA sequencing studies have revealed distinct immune responses in patients with the Δ382 SARS-CoV-2 variant as compared to wildtype SARS-CoV-2, including enhanced adaptive immune responses, improved T cell functionality, robust SARS-CoV-2-specific immunity, and rapid antibody production [160]. Another study identified SARS-CoV-2-induced DNA methylation changes, with significant downregulation of DNA methyltransferases (DNMT1, DNMT3A, DNMT3B) leading to promoter hypomethylation and upregulation of genes like HSPA1L and ULBP2. These genes showed increased expression in asymptomatic and severe cases, with HSPA1L implicated in viral replication. These findings highlight the SARS-CoV-2-driven epigenetic modifications as potential therapeutic avenues, though further research with larger patient cohorts is needed to elucidate underlying mechanisms and improve clinical applicability [161].

## 5. Susceptibility to Viral ARIs and Bacterial Co-Infections: Interactions, and Clinical Outcomes

The clinical impact of viral co-infections has been a subject of extensive study, yielding mixed results. Some studies suggest that co-infections can exacerbate disease severity, increasing the risk of moderate to severe disease, non-invasive ventilation, or death. However, other studies report no significant differences or even reduced severity in certain cases [162]. Research indicates that RSV and human rhinoviruses (HRVs) frequently co-occur in children, though viral interference is observed. For example, the detection rate of rhinovirus decreases in the presence of RSV, whereas children receiving RSV immunoprophylaxis are more likely to be infected with human rhinoviruses [163]. Competition among respiratory viruses extends to RSV and influenza, where peaks in one virus’s activity correspond to declines in the other. Notably, co-infection rates between RSV and influenza are unexpectedly low, likely due to competitive interference [164,165,166]. While influenza co-infections with multiple strains increase the risk of ICU admission or death, RSV/rhinovirus co-infections mainly prolong hospital stays without necessarily worsening ICU or ventilation outcomes [167]. Furthermore, systematic reviews and large-scale analyses have often concluded that viral co-infections, particularly in children, do not significantly impact clinical outcomes such as hospital length of stay, ICU admissions, or mechanical ventilation requirements [167,168,169]. Some studies even report less severe illness in co-infected children over three months of age compared to those with single infections [170,171]. Overall, the clinical implications of viral co-infections remain variable, influenced by factors like patient age, viral combinations, and study populations.

The interactions among co-infecting viruses can lead to synergistic or antagonistic effects, often altering cytokine and chemokine expression patterns [172,173]. In murine models, RSV mono-infection induced minimal TNF-α and IL-6 levels, whereas IAV mono-infection significantly elevated these cytokines. Sequential infections revealed that prior RSV infection decreased TNF-α and IL-6 levels following subsequent IAV infection, suggesting protective effects mediated by innate immune responses [174,175]. Similarly, RSV has been shown to inhibit HMPV replication through type I and III interferon-mediated responses, with disruption of these pathways reducing RSV’s inhibitory effects [176,177]. In human airway cells, HRV infection transiently protected against IAV or pH1N1 replication by inducing interferon-stimulated genes [178]. Additionally, co-infections involving IAV and SARS-CoV-2 exhibited cytokine dynamics dependent on the infection sequence, with elevated TNF-α, IL-6, and IFN-β levels following sequential infections, particularly when SARS-CoV-2 preceded IAV [179]. These findings highlight the critical role of interferons and immune modulation during viral co-infections and underscore the complexity of host–pathogen interactions.

Interactions between respiratory viruses and bacteria also are well-documented and frequently contribute to exacerbations of chronic respiratory diseases like COPD. Viral infections, such as rhinovirus, can elevate bacterial burden, as evidenced by increased bacterial 16S rRNA gene levels in COPD patient’s post-infection [180,181]. Influenza infection enhances susceptibility to *S. pneumoniae* and *S. aureus* through mechanisms like impaired macrophage function, diminished TNF-α-induced NK cell activity, and reduced IL-1β and Type 17 immunity [182,183,184,185]. Conversely, *S. pneumoniae* infections can decrease lung CD8^+^ T cell populations and virus-specific antibodies (IgA, IgM, and IgG), along with reductions in CD4^+^ T cells, B cells, and plasma cells, thereby promoting viral persistence and increasing mortality rates [186,187]. Respiratory viral infections also heighten the risk of secondary bacterial pneumonia and amplify bacterial virulence. During acute inflammation, cytokines like IL-1β, IL-6, and TNF-α can foster bacterial growth [188,189]. Viral-bacterial co-infections also contribute to severe outcomes, as seen in RSV-associated bronchiolitis and bacterial superinfections, which correlate with longer hospital stays and higher severity scores [190]. Historically, the 1918 influenza pandemic highlighted the lethal impact of secondary bacterial infections, predominantly bacterial pneumonia. In children, concurrent viral and bacterial infections are common in acute otitis media (OM), with pathogens such as *S. pneumoniae*, *H. influenzae*, and RSV frequently implicated [191,192]. Viral infections impair mucociliary clearance and Eustachian tube function, promoting bacterial colonization and middle ear inflammation [191,193,194]. These insights underscore the complex dynamics of viral-bacterial interactions and their implications for disease severity.

## 6. Diagnostic Applications of Epigenetics in Viral ARIs

### 6.1. Epigenetic Biomarkers

In the genome of an individual, epigenetic changes represent a well-orchestrated symphony for modulation of the transcriptional outcome of the genetic code. Epigenetic biomarkers provide groundbreaking evidence not only in terms of facilitating prognostication and diagnosis of disease and subsequent treatment monitoring but also highlight the importance of nutrition, metabolic states and environmental factors on health. A key feature of epigenetic marks—such as DNA methylation and miRNAs—obtained from diverse biological specimens (e.g., blood, plasma, urine, and formalin-fixed paraffin-embedded tissues) is their remarkable stability, making them reliable indicators for predicting disease risk, aiding diagnosis, and assessing therapeutic outcomes [195,196]. Epigenetic modifications, including DNA and RNA methylation, histone modifications, and ncRNAs, have been implicated in creating perturbations of the transcriptional activity related to host-immune interactions by modulation of chromatin structure and gene expression patterns. These mechanisms play a crucial role in host–pathogen interactions, particularly in severe viral respiratory infections, where epigenetically regulated processes affect innate and adaptive immune responses, inflammation, and viral outcomes. For example, DNA methylation and histone modifications influence host antigen presentation in infections like H5N1 and MERS-CoV [197].

### 6.2. Epigenetic Patterns for Disease Assessment and Risk Stratification

Viruses causing severe respiratory illnesses have been shown to utilize three major epigenetic regulatory mechanisms to influence host–pathogen interactions, namely (i) coding for viral proteins capable of directly interacting with host-modified histones to reprogram chromatin structure and gene expression; (ii) altering DNA methylation patterns and host miRNA expression profiles, thereby dysregulating genes involved in innate and adaptive antiviral immune responses; and (iii) regulating viral lifecycle and host immune responses by hijacking the host’s nuclear miRNA processing machinery responsible for encoding viral non-canonical miRNA-like RNA fragments (v-miRNAs). For example, H5N1 and SARS-CoV-2 have been observed to modulate host epigenetic mechanisms, contributing to susceptibility to pulmonary illnesses by interrupting both innate and adaptive immune pathways. This epigenetic interference enhances viral persistence and pathogenesis in the host, underlining the critical role of these mechanisms in disease susceptibility and progression [198,199,200]. Some relevant mechanisms are discussed below in relation to common respiratory viral diseases.

#### 6.2.1. Histone Modifications and Viral miRNAs in Influenza A Virus

Recent multi-omics studies have highlighted the ability of avian Influenza A virus (H5N1) to evade early host antiviral responses through modifications of histone methylation patterns at type I interferon-sensitive genes (ISGs). Specifically, in human airway epithelial cells, the H5N1 virus induces the formation of heterochromatin state at genomic regions adjacent to SMAD9L, CFHR1, and DDX58 genes. This chromatin state is characterized by an increase in the repressive histone mark H3K27me3 and a concomitant decrease in the active histone mark H3K4me3, mediated by the NS1 viral protein [31]. In the viral life cycle, small viral leader RNAs (v-miRNAs) play a crucial role in producing new progeny virions by facilitating genomic RNA encapsidation. These v-miRNAs are encoded by the 5′ ends of all eight genomic segments of the Influenza virus [201]. Studies have shown that the H5N1 virus encodes miR-HA-3p, a miRNA-like small RNA that plays a role in increasing the production of antiviral cytokines in human macrophages. This molecule serves as an important virulence marker, indicating the severity of the H5N1-induced cytokine storm and its associated high mortality rates. Furthermore, the viral machinery responsible for producing functional miRNAs can be harnessed to develop miRNA delivery systems using RNA viruses as molecular vectors [202,203,204].

#### 6.2.2. DNA Methylation and NETosis in SARS-CoV-2

Compared to earlier COVID viruses, the increased transmissibility rates and asymptomatic infection states of SARS-CoV-2 can be attributed to a highly efficient replication machinery coupled with lower IFN production in lung tissues [205]. Studies have shown that epigenetic pathways modulated by oxidative stress lead to ACE2 deregulation. In systemic lupus erythematosus patients, CpG hypomethylation of the ACE2 gene is conjectured to increase susceptibility to COVID-19 and exacerbate its severity by upregulating ACE2 protein expression in T cells, thereby facilitating viral infection and spread [206]. These epigenetic signatures might favor viral infection and provide risk biomarkers to understand disease severity and progression in SARS-CoV-2 infection. Under physiological conditions, NETosis provides a form of innate immunity, in which histone H3 modifications guide the death of neutrophils, leading to the release of neutrophil extracellular traps (NETs). These NETs serve as a scaffold for platelet aggregation and adhesion, resulting in the entrapment of pathogens and preventing their diffusion. This scaffold comprises a complex of DNA fibers, histones, and other proteins [197,207]. These NETs can stimulate macrophages to secrete IL1B, thereby prolonging the signaling cascade between macrophages and neutrophils and causing progressive inflammatory damage. Previous studies have linked thrombotic events and lung inflammation, as well as extensive lung damage and ARDS, to dysregulation of NETosis [207,208]. Since the pandemic began, it has been observed that COVID-19 patients have a higher predilection for thromboembolic events and disseminated intravascular coagulation [208,209]. Recently, a study by Barnes and colleagues highlighted the significant role of NETosis in neutrophilic lung infiltration, attributing it to organ damage and subsequent mortality in COVID-19 patients [210]. The molecular basis of heparin’s efficacy in reducing mortality in patients with severe COVID-19 symptoms complicated by sepsis-induced coagulopathy is provided by its ability to disrupt NETs and block histone-induced platelet aggregation [211]. In this context, for patients with severe COVID-19, DNase I-mediated degradation of NETs holds promise as a novel therapeutic option [212]. For a better understanding of viral regulation, the epitranscriptome of SARS-CoV-2 was analyzed using nanopore direct RNA sequencing. In this analysis, around 41 RNA modification sites were identified, most commonly present in the AAGAA motif on viral transcripts with shorter poly(A) tails. The presence of these poly(A) tails, which play a crucial role in RNA stability and turnover, may represent one of the plausible molecular mechanisms deployed by SARS-CoV-2 to escape the host immune defenses and enhance the cytokine storm [213].

### 6.3. Evidence of Epigenetic Immune Regulation from Human Studies

The epigenetic profile analysis of blood samples from patients infected with SARS-CoV-2, drawn from a large cohort of young recruits, was conducted as part of the COVID-19 Health Action Response for Marines (CHARM) study. The findings indicated persistent changes in the post-infection DNA methylation profile in blood cells compared to the pre-infection profile, with these changes lasting at least several weeks after infection [16]. The characteristic epigenetic signature observed in the post-infection state prominently featured an increased proportion of hypomethylated regions in close proximity to ISGs, which showed significant overlap with epigenetic signatures previously identified in autoimmune diseases such as multiple sclerosis and systemic lupus erythematosus [157]. An independent study examining the epigenetic responses in blood samples from eight convalescent individuals (4–12 weeks post-recovery) using single-cell ATAC-sequencing assays demonstrated distinct chromatin accessibility profiles, particularly in CD14^+^ and CD16^+^ monocytes, compared to healthy individuals [214]. However, another study, which employed DNA methylation analysis, single-cell RNA-seq, and single-cell ATAC-seq methods to investigate the epigenetic responses in recovering COVID-19 patients, revealed only limited differences between controls and convalescent participants [215]. These studies collectively underscore that long-term epigenetic changes in the immune system can result from SARS-CoV-2 infection [216]. However, the extent or specific consequences of these post-infection changes in epigenetic signatures, linked to immune system perturbations, remain incompletely understood. Such alterations have been broadly categorized as “long COVID” or Post-Acute Sequelae of SARS-CoV-2 infection (PASC) [217]. It can however be conjectured that PASC can be attributed to some form of virus-induced epigenetic modification, a premise warranting further investigation. Similar long-term respiratory sequelae leading to asthma, allergies, and other pulmonary conditions, have also been observed in children infected with RSV [218,219,220,221]. This is further supported by a recent study conducted to characterize the DNA methylation profile of blood samples from children under 2 years old diagnosed with RSV infection, and this monitoring was conducted for at least 3 years to observe the development of asthma or recurrent wheezing [132] which can be attributed to the alteration of DNA methylation patterns in immune cells.

Epigenetic regulation of pulmonary viral infections is also mediated by lncRNAs, as evidenced in human studies. Hundreds of lncRNAs with varying expression levels have been identified in patients diagnosed with IAV and SARS-CoV-2 infections compared to negative controls. Notably, one lncRNA, named CHROMR, which is associated with lipid metabolism, exhibits distinctive histone acetylation patterns at the regulatory regions of ISGs and plays a crucial role in restricting viral infections in macrophages [222].

### 6.4. Evidence of Epigenetic Immune Regulation from In Vitro and Animal Models

In addition to human studies, significant insights have been provided by animal and in vitro models, illustrating the impact of IAV, SARS-CoV-2, and RSV infections on remodeling the innate immune system via epigenetic changes. One important mechanism through which these respiratory viruses influence epigenetic signatures is the upregulation or downregulation of enzymes such as HDACs and DNMTs. In a study where blood cells from healthy human donors were exposed in vitro to either the H1N1 or H5N1 strain of IAV, an upregulation of epigenetically relevant enzymes, including TET1, TET3, DNMT1, DNMT3A, and DNMT3B, was observed. Among these strains, H1N1 not only increased TET2 expression and overall DNA demethylation levels but also decreased the expression of UHRF1, a key epigenetic regulator that binds to DNA and promotes methylation by recruiting DNMT1 [223]. Similarly, SARS-CoV-2 infection in human epithelial cells in vitro demonstrated downregulation of DNMT1, DNMT3A, and DNMT3B enzymes [161]. Additionally, IAV has been shown to downregulate HDAC1 and HDAC2 gene expression in vitro, which correlates with increased viral replication [224,225]. Although these studies demonstrate that infections can alter the expression of enzymes responsible for regulating DNA methylation or histone modifications, the underlying mechanisms driving these alterations remain poorly understood. IAV infection is also known to cause histone modifications, as evidenced by a decrease in histone acetylation in IAV-infected A549 cells, which impaired cellular transcription [13]. Furthermore, in a mouse model, IAV infection was found to upregulate the gene expression of the methyltransferase Setdb2 in myeloid cells within the lungs, an effect typically induced by type I interferon signaling. Similarly, RSV infection alters the histone methylation machinery, as demonstrated in a mouse model where RSV increased the expression of Jmjd3 and Utx H3K27 demethylases in bone marrow-derived dendritic cells and lung dendritic cells [226].

## 7. Therapeutic Interventions Targeting Epigenetic Mechanisms

In order to combat modern drug-resistant pathogens, the discovery of novel drugs or the repurposing of already approved drugs is imperative [227]. Drugs targeting the epigenetic pathways, or “epi-drugs,” offer a promising therapeutic strategy for regulating viral-host interactions during critical illnesses [228]. The advent of SARS-CoV-2 further motivated researchers to explore the repurposing of FDA-approved epi-drugs [229] as well as commonly used drugs such as metformin and statins. Recently, a few epi-drugs, such as vorinostat and belinostat, have been introduced into clinical use primarily for the treatment of hematological malignancies. Many other epigenetic-based drugs are currently undergoing trials to validate their potential for mainstream or adjuvant therapy against infectious diseases (Table 3).

### 7.1. Epidrugs

Based on their respective target enzymes, epidrugs are classified into the following categories: histone deacetylase inhibitors (HDACi/KDACi), histone acetyltransferase inhibitors (HATi/KATi), histone N-methyl lysine demethylase inhibitors (HDMi/KDMi), DNA N-methyltransferase inhibitors (DNMTi), histone methyltransferase inhibitors (HMTi/KMTi), and bromodomain inhibitors. At present, two classes of epigenetic drugs, namely DNMTi and HDACi, have been approved by the FDA for clinical use, while other classes are under clinical trials [230]. Azacitidine, the first approved DNMTi, is currently used for chronic myelomonocytic leukemia and myelodysplastic syndrome. Apart from the two approved epidrug classes, HMTi and bromodomain inhibitors are emerging as promising epidrug classes. Bromodomain proteins are specialized reader proteins that recognize acetylated lysine residues, facilitating gene activation through signal transduction [231]. OTX-015 and CPI-0610, both targeting Bromodomain Extra-Terminal (BET) proteins are examples of bromodomain protein inhibitors being used in phase I cancer trials [232].

**Figure 2:**
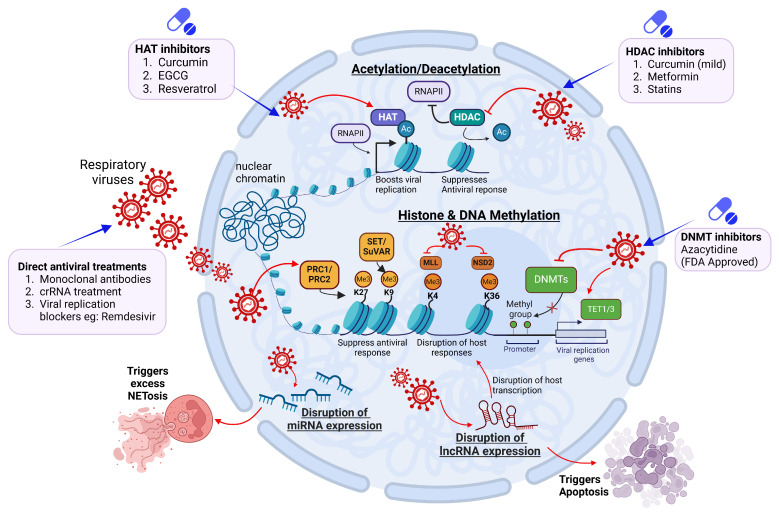
**Impact of respiratory viral infections on epigenetic modifications and therapeutic interventions:** This figure illustrates how respiratory viral infections influence key epigenetic modifications, including histone acetylation, histone methylation, DNA methylation, and RNA-based transcriptional regulation, alongside potential therapeutic strategies targeting these pathways. Viral infections modulate histone acetylation within the nucleus (via histone acetyltransferases [HATs] and histone deacetylases [HDACs]), with acetylation promoting transcriptional activation and deacetylation suppressing antiviral responses. They also alter histone methylation through enzymes like PRC1/PRC2 and SET/SuVAR, where repressive marks (e.g., H3K27me3) suppress antiviral genes, and active marks (e.g., H3K4me3) are dysregulated during viral infections. DNA methyltransferases (DNMTs) silence promoter regions of antiviral genes, while TET enzymes regulate DNA demethylation to restore immune gene expression. RNA-based regulation, including miRNA disruption and lncRNA dysregulation, interferes with transcriptional responses, while excessive NETosis exacerbates inflammation. Therapeutic interventions include epigenetic modulators such as HAT inhibitors (e.g., curcumin), HDAC inhibitors (e.g., metformin, statins), DNMT inhibitors (e.g., azacitidine), and direct antiviral treatments (e.g., monoclonal antibodies, CRISPR-based therapies, remdesivir) (Created with BioRender.com).

Among the de novo epidrugs, both curcumin and apabetalone have shown potential therapeutic benefits. Curcumin, a natural polyphenol derived from turmeric, is a known HDACi. Studies have demonstrated that curcumin treatment can downregulate the secretion of pro-inflammatory cytokines during H1N1 infection and decrease the expression of NF-κB gene in human macrophages [233]. This mechanism suggests that curcumin may be effective in mitigating IAV-induced severe lung infections by downscaling of cytokine signaling without compromising immune function. Notably, curcumin and its derivative demethoxy-curcumin have been shown to possibly inhibit key proteases of SARS-CoV-2, which are crucial for viral replication and transcription. Apabetalone, a direct inhibitor of BET (Bromodomain and Extraterminal) 2/4 protein-SARS-CoV-2 interactions, may suppress the expression of ACE2 receptors—key cellular entry points utilized by the SARS-CoV-2 surface S glycoprotein [234]. Although apabetalone is not yet FDA-approved, it has demonstrated safety and efficacy in phase III trials, such as the BETonMACE trial, which targeted secondary prevention of cardiovascular dysfunction in diabetic patients [235]. These findings suggest that apabetalone may serve as a promising therapeutic agent for inhibiting viral replication and managing infections. In this context, we have presented a schematic representation that illustrates the intricate interplay among respiratory viral infections, epigenetic regulation, and potential therapeutic strategies targeting these pathways (Figure 2).

### 7.2. Repurposed Drugs with Antiviral Properties

Certain drugs with well-established applications have been repurposed as epidrugs to manage respiratory viral infections. Among these, statins and metformin have shown promising antiviral and anti-inflammatory properties. Statins, chemically classified as hydroxymethylglutaryl (HMG) coenzyme A reductase inhibitors, are primarily used as cholesterol-lowering drugs. However, they also exhibit pleiotropic epigenetic effects, including the inhibition of HDACs. Having demonstrated anti-inflammatory effects [236], statins were also expected to inhibit the cytokine storm induced by influenza viruses. Studies have highlighted the role of statins in modulating various molecular pathways associated with the viral lifecycle, thereby offering potential therapeutic options for influenza, primarily through the reduction in apoptosis [237,238]. Experimental studies have also conjectured the use of early and high dose of statins as a beneficial strategy for treating MERS-CoV infections by directly targeting the Toll-like receptor (TLR)-MYD88-NF-κB axis, a pathway implicated in severe respiratory infections, including SARS-CoV-2 [239,240,241]. This regulatory role of statins in the MYD88 pathway may provide a promising avenue for enhancing innate immune responses and limiting viral respiratory infections. Metformin, a widely used first-line anti-hyperglycaemic drug for type 2 diabetes (T2D), is also identified as an HDAC inhibitor. Beyond its glucose-lowering effects, metformin modulates inflammatory pathways to reduce chronic inflammation. Furthermore, it has shown potential to improve the immune response to the influenza vaccine by enhancing B-cell function, downregulating inflammatory responses, and upregulating AMPK phosphorylation [242].

### 7.3. Off-Target Effects of Epidrugs

The off-target effects of epidrugs often limit their utility and lead to their restricted use. Different classes of epidrugs present unique challenges and need to be carefully monitored to prevent unwanted side effects in the treated patients. The commonly used HDAC inhibitors have been demonstrated to disrupt both genome stability and DNA repair through several mechanisms. Researchers have shown that DNA damage can occur due to the stalling of replication forks induced by vorinostat, with similar effects observed following HDAC3 knockdown. These outcomes are primarily attributed to aberrant firing of the replication origin mediated by chromatin opening induced by HDAC inhibitors [243,244]. Additionally, HDACi have been found to downregulate DNA repair genes, leading to enhanced radiosensitivity of cells [245]. Another study showed that HDACi had the potential to modulate DNA repair through direct deacetylation of some of the DNA repair proteins such as Ku70 and PARP1 [246]. Another drug, Entinostat, when used at high concentrations, has been shown to induce ROS within 2 h of treatment, primarily through the loss of mitochondrial membrane potential [247]. Another group of epidrugs, histone acetyltransferase (HAT) inhibitors, also demonstrated off-target effects. These effects are largely due to their reduced specificity, which can adversely affect non-target proteins and influence cell viability even at low concentrations when the drug is not directly targeting the intended HAT. Such non-specific acetylation patterns can lead to changes in gene expression, disrupt normal cellular processes unrelated to histone modification, and cause adverse effects in normal cells [248]. On the other hand, epidrugs belonging to the DNMTi family have been shown to cause unintended changes in DNA methylation patterns at sites not specified by the target genes, leading to unwanted side effects. These effects may manifest as altered gene expression patterns in non-cancerous cells, affecting normal cellular functions leading to toxicity along with reversal of the covalent protein-DNA linkages, resulting in the dissociation of the enzyme from the DNA. For example, drugs such as decitabine, 5-Aza, and zebularine, which cause substitutions at the target cytosine, interfere with the reaction cycle. This interference can lead to sustained and/or irreversible DNA cytosine-C5 methyltransferase (MTase)-DNA adducts [249]. Researchers have demonstrated the cytotoxic mechanisms of DNMT inhibitors through in vivo studies, which suggest that (a) decitabine cytotoxicity is mediated via the formation of protein-DNA adducts [250]; (b) cytotoxicity levels are directly proportional to MTase levels [251]; and (c) MTase-DNA adducts induced by decitabine activate the p53 DNA damage response [252,253,254,255]. The off-target effects of HMTi drugs arise from unwarranted changes in gene expression patterns in non-cancerous cells, leading to side effects that affect physiological processes such as cellular differentiation, development, and immune function [256]. HMTs utilize S-adenosyl methionine (SAM) as the methyl group donor to target lysine and arginine residues present on histone proteins, exerting various regulatory effects [257]. Experiments in mouse models have shown that excision of the methyltransferase active domain (SET domain) in the MLL (Mixed Lineage Leukemia) gene results in defects in skeletal development as well as abnormal expression of Homeobox-related genes [258]. Bromodomain inhibitors, particularly BET (Bromodomain and Extra-Terminal) inhibitors, exhibit off-target effects when the small molecule drug binds to unintended protein targets in addition to bromodomains. This results in unwarranted cellular effects, manifesting as hematological side effects (e.g., anemia and thrombocytopenia), gastrointestinal issues, as well as alterations of cellular functions resulting from transcriptional changes.

Interestingly, these off-target effects can sometimes have positive serendipitous outcomes. Recent studies on clinical-stage kinase inhibitors have highlighted that the JAK2 inhibitor TG101209, the CDK inhibitor dinaciclib, and the PLK1 inhibitor BI-2536 significantly inhibit BET bromodomains as an unintended off-target effect, potentially contributing to their therapeutic efficacy [259,260,261,262]. These findings suggest that rational drug design targeting both specific kinases and BET proteins can further enhance anticancer therapy and prevent therapeutic resistance [263].

### 7.4. Immunomodulators as Antivirals: Insights from COVID-19

During the COVID-19 pandemic, tocilizumab (TCZ), a humanized monoclonal antibody, emerged as one of the important repurposed drugs undergoing clinical trials for the treatment of critically ill pneumonia patients. Tocilizumab is known to reduce cytokine storms by inhibiting interleukin-6 (IL-6) receptor signaling, which is associated with an increased risk of cardiovascular mortality [264]. Several older-generation anti-inflammatory drugs, including sarilumab (NCT04315298; Phases 2 and 3) and TCZ (NCT04320615; Phase 3), were also put into clinical trials. Additionally, established antiviral agents with known mechanisms for blocking viral replication, such as favipiravir (NCT04358549; Phase 2) and remdesivir (NCT04292730; Phase 3), were evaluated in clinical trials compared to standard therapeutic procedures. Reports have pointed out that the clinical effects of intravenously administered remdesivir seems to be at the most modest, contrary to preliminary expectations. However, a randomized Phase 3 clinical trial (NCT04292899) demonstrated not statistically significant difference in the efficacy between 5- and 10-day courses of remdesivir compared to standard treatment [265].

### 7.5. CRISPR/Cas9 and Epigenome Editing

In the modern era, several genome editing technologies, particularly CRISPR/Cas9, transcription activator-like effector nucleases (TALENs), and zinc-finger nucleases, have been developed for highly specific editing of targeted genomic sequences [266]. Scientists have also developed a CRISPR/Cas13-based technology called PAC-MAN for viral inhibition that can efficiently degrades RNA from SARS-CoV-2 sequences and the live IAV. In this approach, CRISPR RNAs (crRNAs) were elegantly designed and screened to specifically target conserved viral regions, facilitating the identification of functional crRNAs for SARS-CoV-2 targeting [267]. Bioinformatic analysis from this study identified a set of six crRNAs capable of efficiently targeting more than 90% of all *Coronaviridae*. Another group of researchers also developed a CRISPR/Cas13-based system targeting SARS-CoV-2 by designing crRNAs specific to the nucleocapsid and replicase genes of SARS-CoV-2 which halted viral replication and alleviated symptoms in a hamster model [268]. Additionally, a type III CRISPR-based RNA editing system, named TEAR-CoV, was also discovered to combat SARS-CoV-2, which demonstrated efficacy in both in vitro studies as well as in eukaryotic cells [269]. Along with SARS-CoV-2, Blanchard et al. also developed a CRISPR/Cas13-based system to combat the influenza virus by designing crRNAs specific to IAV regions such as PB1 and highly conserved regions of PB2. Their findings showed that selected crRNAs and the Cas13a protein effectively reduced viral RNA levels in cell cultures. Furthermore, in mouse models, Cas13a-mediated degradation of influenza RNA significantly decreased viral levels in lung tissues [268].

### 7.6. Role of Precision Medicine in Targeting SARS-CoV-2

Since the COVID-19 pandemic, it has become evident that precision medicine has advanced significantly due to numerous biological discoveries and the rapid progression of high-throughput multi-omics technologies. Scientific advancements in omics technologies, such as metabolomics/lipidomics, microbiomics, proteomics, transcriptomics, genomics, and epigenomics, have become pillars of precision diagnostics. The vast amounts of data generated from various omics technologies have facilitated the identification and analysis of omics-based biomarkers, which in turn become the principal driving force in advancing precision and preventive medicine. Precision medicine focuses on early disease diagnosis, monitoring disease prognosis, tailoring individualized treatments, and offering suitable vaccination strategies [270]. Precision therapies for COVID-19 have traditionally been divided into two major categories, namely (1) therapies targeting the SARS-CoV-2 virus directly, and (2) therapies aimed at modulating host immune responses. It is quite apparent that the effective therapy to halt disease progression relies on the effectiveness of both drug groups; and hence, multiple combinations of drugs have been tested. For instance, remdesivir, the first FDA-approved antiviral for COVID-19, reduces disease progression, and improves post-hospitalization outcomes in both elderly and pediatric patients [271,272]. Similarly, a phase 3 randomized, placebo-controlled trial with 1433 COVID-19 participants found that oral molnupiravir was more effective during the pre-hospitalization stage [273]. However, in urgent cases, ritonavir-boosted nirmatrelvir has also been prescribed to older adults (aged > 65 years) or patients with comorbidities, such as diabetes, obesity, cardiovascular disease, or cancer [274]. Apart from precision and combinatorial therapies, the future of precision medicine will heavily rely on further advancements in omics technologies, particularly focusing on single-cell multi-omics data integration to enable complex multi-level analyses of intracellular signalosome profiles. Moreover, emerging artificial intelligence (AI) technologies, enhanced by advanced neural networks, are expected to amplify evidence-based insights derived from multi-omic and clinical data, which will be crucial for driving the evolution of precision medicine and improving public health outcomes.

## 8. Discussion: Challenges and Future Perspectives

As research continues to unveil the significance of epigenetic mechanisms in viral ARIs, novel opportunities for diagnostics and therapeutics are emerging. However, translating these findings into clinical applications remains challenging due to technical complexities and methodological limitations. Epigenetic modifications such as DNA methylation, histone modifications, chromatin remodeling, and ncRNA activity play critical roles in regulating immune responses and viral life cycles [275,276]. Viruses like SARS-CoV-2, RSV, and Influenza A exploit host epigenetic machinery to alter gene expression, facilitating replication and evading immune detection [277]. For instance, SARS-CoV-2 ORF8 mimics histone ARKS motifs, disrupting chromatin organization and inhibiting immune activation by limiting chromatin accessibility [158]. These epigenetic manipulations have implications for long-term host immune memory, reinfection, and chronic disease. Epigenetic profiling offers diagnostic potential, with approaches like reduced representation bisulfite sequencing (RRBS) and whole-genome bisulfite sequencing (WGBS) accurately identifying hyper- and hypomethylation patterns, even in blood or respiratory epithelium [278]. Implementing these methylation patterns into diagnostic systems would make it possible to detect viruses early and distinguish between non-viral ARIs. Viral infections often induce hypomethylation of ISGs, and hypermethylation of suppressive regulatory elements, which could serve as diagnostic signatures [157]. Moreover, techniques such as chromatin immunoprecipitation followed by sequencing (ChIP-seq) enable detailed profiling of histone modifications, providing valuable insights into viral replication and host immune responses [279,280,281].

Reversible epigenetic changes hold significant potential for precision medicine, with small-molecule inhibitors originally developed for chromatin remodeling in cancer now being explored for their potential to restore immune function during viral infections [282]. Viruses exploit chromatin remodelers to evade immune defenses, disrupting interferon and pro-inflammatory cytokine responses critical for controlling ARIs. These disruptions can also have long-term effects, impairing immune memory and increasing vulnerability to reinfections. Therapies targeting chromatin remodeling complexes, such as DNA methyltransferase inhibitors (e.g., 5-azacytidine) and histone deacetylase inhibitors (e.g., vorinostat), show promise in modulating immune responses by restoring chromatin accessibility and antiviral gene expression [283,284]. New therapies, such as CRISPR-based epigenome editing, have emerged as potential tools to selectively activate or silence gene expression. However, precise delivery to infected cells remains a significant challenge [285,286]. Epigenetic-targeted treatments offer huge promise but are both technically and clinically challenging. For example, targeted delivery of DNA methyltransferase inhibitors or histone deacetylase inhibitors to infected cells is challenging, as off-target effects may disrupt critical cellular functions [287,288]. To address these limitations, drug delivery systems like nanoparticle carriers are being investigated for their potential to enhance tissue specificity and reduce systemic toxicity. However, these systems remain in the experimental stage for applications in ARIs [289,290]. Moreover, while small-molecule inhibitors are efficient, they may lack specificity in viral infection settings and could inadvertently silence host genes essential for normal cellular functions. CRISPR-based epigenome editing offers a highly specific method to modulate gene expression, with the use of tools such as dCas9 combined with epigenetic effectors (e.g., dCas9-TET1 for DNA demethylation). These systems can be directed to genomic loci critical for antiviral responses, potentially reinvigorating host antiviral mechanisms [291,292]. Nonetheless, significant hurdles persist in efficiently delivering CRISPR complexes to respiratory cells in vivo. Moreover, the short-term use of CRISPR devices to minimize immune attack and off-target effects remains an area of active investigation. Continued research and technological innovation are essential to overcome these challenges and unlock the full therapeutic potential of epigenetic-targeted interventions in viral infections.

Although epigenetic research holds significant potential for understanding and managing viral ARIs, technical barriers in ARI research constrain both limited sample sizes and reproducibility issues which present significant challenges. The high-throughput identification and quantification of molecular epigenetic signatures during infection relies on advanced sequencing and bioinformatics technologies. Whole-genome sequencing, while powerful, is cost-intensive, generates vast amounts of data, and requires sophisticated bioinformatics pipelines to identify and analyze specific methylation patterns. In addition, defining primary epigenetic changes induced by viral infection and secondary epigenetic alterations induced by host immune responses are tricky and requires time-course studies and single-cell analyses to make a definitive distinction. Emerging techniques like single-cell ATAC-seq (scATAC-seq) and single-cell RNA-seq (scRNA-seq) are promising techniques for characterizing cell-type-specific epigenetic and transcriptional signatures within heterogenous tissues such as the lungs [293,294]. However, these approaches remain technically challenging and require further optimization to elucidate epigenetic changes in the dynamic context of viral infections. To ensure reliability and comparability across studies, standardized protocols for sample collection, handling, and analysis must be established.

Studies on DNA methylation and histone modifications have provided valuable insights into ARIs, but many critical questions remain unanswered, particularly regarding how chromatin remodels during infection and the roles ncRNAs in viral pathogenesis. By regulating immune pathways at both transcriptional and post-transcriptional levels, ncRNAs enable viruses to evade host defenses and establish persistent infections. Measuring specific ncRNA levels in patients could help predict disease severity, and therapeutics targeting ncRNA activity hold promise for limiting viral replication while enhancing antiviral immunity. One therapeutic approach involves targeting pro-viral miRNAs to inhibit their function, thereby restoring the host’s antiviral response. MiRNA inhibitors designed to specifically block miRNAs that promote viral replication and show potential in combating viral infections [295,296]. A specialized class of miRNA inhibitors, known as antagomirs, is being evaluated in clinical trials for their ability to neutralize specific miRNAs, potentially reducing viral load and enhancing immune function. By preventing pro-viral miRNAs from hijacking host cellular machinery, antagomirs could enable a more robust immune response [297]. Given their significant roles in modulating immune responses, ncRNAs represent promising therapeutic targets and potential biomarkers for predicting disease severity in viral ARIs [53,59]. Moreover, advancements in AI and machine learning could also offer opportunities to analyze large-scale epigenetic datasets, potentially uncovering predictive biomarkers for disease severity and therapeutic outcomes [298]. Further improving the specificity and efficacy of epigenetic therapies will depend on innovations in drug delivery technologies, such as lipid nanoparticles and viral vectors, which could enhance tissue targeting and minimize off-target effects. Overall, integrating sophisticated epigenetic tools, computational methods, and targeted therapies opens a promising new frontier to treat viral ARIs in a more personalized and effective way. Despite these advancements, few studies have also addressed how epigenetic variations in specific virus strains or host cells influence the course of infection or therapeutic efficacy [299]. Expanding epigenetic research to encompass a broader range of viral strains, host cell types, and longitudinal post-recovery surveillance could provide deeper insights into the enduring consequences of viral ARIs [300]. Future efforts should prioritize creating detailed virus-specific epigenetic maps and employing multi-omics strategies, including transcriptomics, proteomics, and metabolomics, to provide a comprehensive understanding of host–virus interactions. Technologies like single-cell multi-omics and spatial transcriptomics could enable the monitoring of epigenetic and gene expression fluctuations at single cell level within infected tissues [301]. Longitudinal studies are also essential to determine the persistence of viral-induced epigenetic modifications and their implications for immune memory, reinfection risks, and chronic respiratory syndromes [302]. All these combined approaches will be critical to advancing our understanding and treatment of viral ARIs.

The role of epigenetic modifications in combating viral ARIs continues to draw considerable attention, with innovative strategies offering new insights into viral mechanisms and therapeutic potential. Among these emerging approaches, G-quadruplexes (G4s) have also garnered substantial interest due to their regulatory roles in viral replication, transcription, translation, and epigenetic modulation at various levels. G4s are non-canonical, four-stranded secondary structures composed of guanine-rich nucleic acid sequences, stabilized by Hoogsteen hydrogen bonds. These unique structures have been identified across prokaryotic and eukaryotic genomes and in numerous Baltimore virus groups, including those responsible for viral ARIs [303,304,305,306,307,308]. The functional significance and presence of G4s in most of the viral genomes and their potential as therapeutic targets makes them an attractive focus for antiviral strategies. Accruing evidence has underscored the utility of G4 specific small molecules designed to selectively bind and stabilize viral G4s. These molecules, along with specific G4 interacting proteins that influence the stability of G4 structures, hold promise for novel G4-mediated epigenetic therapeutic interventions [305,309]. However, despite these advances, G4-mediated therapeutic approaches face notable challenges. One of the major challenges includes the development of site-specific and efficient G4-binding molecules that selectively target viral genomes without disrupting host cellular machinery [303,305]. Future studies that integrate structural, biochemical, and pharmacological insights could help refine the therapeutic potential of G4-based strategies and expand our understanding of their roles in viral pathogenesis and treatment.

Taken together, epigenetics offers significant potential for advancing the diagnostic and therapeutic landscape of viral ARIs. However, translating these insights into effective treatments poses substantial technical, methodological, and biological challenges. Continued interdisciplinary research and innovation will be essential to overcome these barriers and develop tangible interventions that improve patient outcomes and mitigate the global burden of viral respiratory infections.

## Figures and Tables

**Table 1:** Epigenetic modifications specific to respiratory viruses, their associated symptoms, and severity implications.

Respiratory Virus	Epigenetic Modifications	Symptoms/Severity Implications	Severity of Disease
Influenza A Virus (IAV)	Histone acetylation of NP (Lys-31, Lys-90 by GCN5/PCAF). Interaction with HDAC1 (Lys-103 acetylation). m6A modifications (IFN-β mRNA stability).	Suppressed interferon responses lead to moderate symptoms in mild cases. Hyperinflammation in severe cases results in lung damage.	Moderate to severe; hyperinflammation in severe cases, cytokine storms.
Respiratory Syncytial Virus (RSV)	DNA methylation changes (e.g., PRF1 enhancer, NODAL gene). H3K4 demethylation by KDM5B. H3K4 methylation by SMYD3 in Tregs.	Weak antiviral responses result in more severe symptoms in infants and immunocompromised individuals. Excessive inflammation contributes to respiratory distress.	Severe in infants and immunocompromised; chronic airway remodeling possible.
SARS-CoV-2	Histone mimicry by ORF8 (chromatin compaction). DNA methylation changes (ACE2 gene hypomethylation).	Dysregulated immune responses cause severe symptoms in COVID-19. Cytokine storms and ARDS are linked to histone acetylation changes.	Mild to life-threatening; ARDS and cytokine storms in severe cases.
Adenovirus	Interaction of protein VII with host nucleosomes. Chromatin condensation by p300-E1A-RB1 complexes.	Moderate respiratory symptoms due to efficient immune evasion. Persistent infections may occur in immunosuppressed individuals.	Moderate; severe outcomes in immunocompromised individuals.
Human Rhinovirus (HRV)	DNA methylation changes at asthma-related genes (e.g., BAT3, NEU1).	Prolonged respiratory symptoms in asthmatics. Contributes to asthma progression by altering immune gene expression.	Mild to severe in asthmatics; prolonged symptoms and asthma exacerbation.
Human Metapneumovirus (HMPV)	m6A modifications in viral genome and mRNA.	Enhances viral replication and gene expression. Increases infection severity via pro-viral effects of m6A-binding proteins.	Moderate to severe; enhanced replication and immune evasion contribute to severity.

**Table 2:** Viral protein interactions with host epigenetic machinery.

Viral Pathogen	Viral Protein	Epigenetic Mechanism	Host Impact
Adenovirus	E1A, Protein VII, E4orf3	Inhibits HDACs Alters histone acetylation at K9 and K18	Increases acetylation, downregulating immune genes while upregulating cell cycle genes for replication
Human Bocavirus (HBoV)	NS1	Influences histone methylation and acetylation, alters the activity of epigenetic modifiers	Involved in viral DNA replication, interacts with various cellular proteins, suppression of antiviral responses
Human Metapneumovirus (HMPV)	G protein	Induces DNA methylation in inflammatory and antiviral genes	Suppresses IFN-stimulated genes, impairing antiviral defenses
Influenza Virus	NS1	Reduces histone acetylation and H3K79 methylation at immune gene promoters	Silences IFN responses, increasing cytokine storms
MERS	ORF4a, Spike protein	Promotes histone deacetylation and H3K27 methylation	Suppresses antigen presentation and immune recognition, aiding persistence
Parainfluenza Virus	C protein	Inhibits histone acetylation	Reduces pro-inflammatory cytokines and IFN signaling to evade immunity
RSV	NS1, NS2	Induces chromatin remodeling	Modulates ECM and TGFβ signaling, reducing IFN production and inflammation
Rhinovirus	VP1, VP4	Alters DNA methylation at immune gene promoters	Links to asthma development and chronic inflammation
SARS-CoV-2	ORF8, Spike protein	Modifies inflammatory gene methylation and ACE2 histone acetylation (H3K4me3, H3K27ac)	Increases ACE2 expression, triggers cytokine storms, and reprograms immune cells epigenetically

**Table 3:** Common epigenetic drugs (epidrugs) in use or under clinical trials for treating acute respiratory infections (ARIs).

Name of Drug	Type/Category	Mode of Action	Effective Against Virus	Effect of Modulation of Epigenetic Pathways	Pro-Inflammatory Cytokine Target If Any	NCT or Trial Number
Apabetalone	De novo epidrug	BET2/4i	SARS-CoV-2	Decreased viral replication	ACE2	-
Curcumin	De novo epidrug	HDACi	Influenza	Decreased inflammation	NF-κβ	-
Metformin	Repurposed epidrug	HDACi	Influenza	Increased antibody response to vaccine	TNF-α	-
Statins	Repurposed epidrug	HDACi	Influenza; MERS-CoV	Reduced viral infectivity	RANTES TLR-MYD88-NF- κβ axis	NCT02056340
Ruxolitinib with simvastatin	Repurposed epidrugs in combination	HDACi + JAKi	SARS-CoV-2 pneumonia	Inhibition of viral entry and anti-inflammatory	RANTES and JAK pathway	NCT04348695
Toclizumab	Repurposed immunomodulatory drug	Not clearly defined mode of epigenetic action	SARS-CoV-2	Reducing inflammation by modulating NETosis	Il-6	NCT04346355 NCT04412772 NCT04424056
Bevacizumab	Repurposed immunomodulatory drug	-	Severe pneumonia of SARS-CoV-2	Prevent ARDS and suppress pulmonary edema	VEGF	NCT04348695 NCT04305106

Abbreviations: ACE2, angiotensin-converting enzyme 2; BETi, bromodomain and extra-terminal domain (BET) inhibitor; HDACi, histone deacetylase inhibitor; IL-6, interleukin-6; MYD88, myeloid differentiation primary response 88; NF-kB, nuclear factor kappa-light-chain enhancer of activated B cell; RANTES, regulation upon activation normal T cell expressed or secreted (CeC chemokine ligand 5); TLR, Toll-like receptor; TNF- α, tumor necrosis factor-alpha; VEGF, Vascular endothelial growth factor.
